# Impact of Primary Care Provider's on Dementia Symptom Management and Healthcare Use

**DOI:** 10.1002/alz70860_107209

**Published:** 2025-12-23

**Authors:** Sidra Haye

**Affiliations:** ^1^ University of Southern California, Los Angeles, CA, USA

## Abstract

**Background:**

The majority of initial diagnosis and post‐diagnosis care is provided by Primary Care Providers (PCPs). In addition to conducting cognitive assessments, referring patients to specialists, helping decide goals of care, PCPs play an integral role in managing medications for persons living with dementia. PCPs manage drugs for dementia symptoms (including donepezil, galantamine, rivastigmine, and memantine) and drugs for behavioral symptoms (including antipsychotics and benzodiazepines). Yet there is an incomplete understanding of the impact of PCPs clinical practice related to medication management on outcomes of persons living with dementia.

**Objective:**

To examine whether medication management for persons with dementia varies across PCPs, and how it impacts hospitalizations and emergency department use of persons living with dementia.

**Methods:**

To separate the impact of PCPs from other factors that impact healthcare use, we focus on patients who are forced to switch to a new PCP after their original PCP exits. We use difference‐in‐differences models using the exogenous variation caused by timing of PCP exit to analyze differences in healthcare use across persons living with dementia. The analysis includes 100% of Medicare claims for TM and MA beneficiaries who were 65 years and over and who were continuously enrolled in Part D and in either TM or MA. Each beneficiary is assigned to the PCP with whom they have the maximum number of evaluation and management visits in a year.

**Results:**

In 2017, 10,776,723 beneficiaries had an assigned PCP. Out of these, 325,995 beneficiaries were affected by PCP exit where PCP exit is defined as the provider stopped treating Medicare beneficiaries. Around 50.4% of these individuals had a claim for one of the four drugs for dementia symptom management. Ongoing work is examining how switching to a PCP with higher medication managing intensity impacts patient healthcare use including hospitalizations and emergency department visits.

**Conclusion:**

Preliminary results suggest that there is considerable variation in prescriptions for drugs for dementia symptom management and drugs for behavioral systems. Ongoing work is examining PCP level factors associated with this variation and its impact on patient outcomes.

